# SNX25 regulates proinflammatory cytokine expression via the NF-κB signal in macrophages

**DOI:** 10.1371/journal.pone.0247840

**Published:** 2021-03-01

**Authors:** Kazuya Nishimura, Tatsuhide Tanaka, Shoko Takemura, Kouko Tatsumi, Akio Wanaka

**Affiliations:** Department of Anatomy and Neuroscience, Nara Medical University, Kashihara, Nara, Japan; Chang Gung University, TAIWAN

## Abstract

Innate immunity is the first line of defense against bacterial infection and is initiated by macrophages. Sorting nexin 25 (SNX25) is an SNX family member and is reported to negatively regulate TGF-β signaling by enhancing TGF receptor degradation. However, few studies have focused on the relationship between SNX25 and the immune system. We knocked down SNX25 expression in macrophages and examined inflammatory cytokine expression, a hallmark of innate immunity, after lipopolysaccharide stimulation. SNX25 knockdown increased proinflammatory cytokine expression in RAW 264.7 cells. In addition, SNX25 knockdown activated the NF-κB signal by promoting ubiquitination of IκBα. These results suggest that SNX25 inhibits the NF-κB signal and thereby regulates proinflammatory cytokine expression in macrophages.

## Introduction

As the first defense layer, innate immunity is initiated mainly by macrophages and recognizes microbial pathogens with pattern recognition receptors (PRRs) [1]. Among the many PRRs, toll-like receptor 4 (TLR4) is well-studied; it recognizes bacterial lipopolysaccharide (LPS) and is involved in innate immunity against gram-negative bacteria [2]. LPS binding to TLR4 results in activation of the TAK1 protein kinase complex, which in turn activates MAPKs (ERK, p38, and JNK) and IKKβ by inducing their phosphorylation. Phosphorylated MAPKs then activate activated protein-1 family transcription factors to regulate inflammatory responses. Activated IKKβ phosphorylates IκBα, which is then degraded by the ubiquitin-proteasome system, leading to nuclear translocation of nuclear factor kappa B (NF-κB) to induce proinflammatory cytokine expression [3, 4]. IκBα ubiquitination is mediated by SCF^β-TrCP^, a specific E3 ubiquitin ligase, and is reduced by deubiquitinating enzymes (DUBs) [5]. Although some studies address the regulation of IκBα by SCF^β-TrCP^ and DUBs [6–8], the details of this regulation remain unclear.

SNXs have been identified from mammals through to yeasts [9]. They play key roles in membrane trafficking, cell signaling, membrane remodeling, and organelle motility [10] and some of them are involved in the immune system [11–13]. SNX25 belongs to the SNX family and negatively regulates TGF-β signaling, which is an important component of the immune system [14, 15]. We serendipitously found that a transgenic mouse line had null mutation of SNX25 and showed reduced inflammatory reactions, especially in dermal macrophages (unpublished observation). We also found that SNX25 is widely expressed in the central nervous system [16] and is possibly involved in the circadian rhythm generation [17]. To gain insights into the relationship between SNX25 and macrophages, we examined whether SNX25 modulates responses of the macrophage cell line RAW 264.7 to LPS stimulation, by knocking down SNX25 expression with specific siRNA. SNX25 knockdown increased proinflammatory cytokine expression by activating the NF-κB pathway via ubiquitination of IκBα. These findings reveal that SNX25 is an important regulator of the TLR4 signaling pathway.

## Materials and methods

### Cell culture

The macrophage cell line RAW 264.7 was cultured in Dulbecco’s modified Eagle’s medium supplemented with 10% fetal bovine serum, 100 U/mL penicillin and 100 μg/mL streptomycin. Cells were grown in a humidified chamber containing an atmosphere of 95% air/5% CO2 at 37°C.

### siRNA transfection of RAW 264.7 cells

SNX25 siRNA-1 (50μM, Sigma-Aldrich, USA, sense; GAUAUUAUGACCAAUCCUU, antisense; AAGGAUUGGUCAUAAUAUC), SNX25 siRNA-2 (50μM, Sigma-Aldrich, sense; CGUACAAUGCUCGCAGAAA, antisense; UUUCUGCGAGCAUUGUACG) was transfected into RAW 264.7 using Lipofectamine RNAiMAX Transfection Reagent (Thermo Fisher Scientific, USA, 13778030) according to the manufacturer’s instructions. Cells were used for experiments 24h after siRNA transfection.

### LPS stimulation

RAW 264.7 cells transfected with siRNA were seeded in 12-well culture plates at a density of 1x10^5^ cells per well and cultured in medium containing 1 μg/ml LPS. Cells were then harvested and were analyzed by Western blotting or RT-qPCR.

### Western blotting

RAW 264.7 cells transfected with siRNA were cultured as described above. For preparation of whole-cell lysates, cells were lysed in radioimmunoprecipitation buffer supplemented with protease inhibitor cocktail (Nacalai Tesque, Japan, L9A7903) and sodium vanadate. The lysates were then sonicated. The homogenate was centrifuged at 20,600 g for 5 min, and the protein concentration in the supernatant was measured using a Micro BCA Protein Assay Kit (Thermo Fisher Scientific). Equal amounts of protein per lane were electrophoresed on SDS-polyacrylamide gels, and then transferred to a polyvinylidene difluoride membrane. After blocking with 5% non-fat milk in phosphate-buffered saline with 0.05% Tween 20 for 1.5 h, proteins of interest were allowed to bind, during an overnight incubation at 4°C, with anti-SNX25 (Proteintech, USA, 13294-1-AP, 1:1000), anti-IL-1β (abcam, UK, ab9722, 1:1000), anti-IL-6 (PeproTech, USA, 500-P56, 1:200), anti-TNF-α (Cell Signaling Technology, USA, #11948, 1:1000), anti-IκBα (Cell Signaling Technology, #9242, 1:1000), anti-phospho-IκBα (Cell Signaling Technology, #9246 1:1000), anti-p65 (Cell Signaling Technology, #8242, 1:1000), anti-phospho-p65 (Cell Signaling Technology, #3033, 1:1000), anti-JNK (Cell Signaling Technology, #9258, 1:1000), anti-phospho-JNK (Cell Signaling Technology, #4668, 1:1000), anti-p38 (Cell Signaling Technology, #9212, 1:1000), anti-phospho-p38 (Cell Signaling Technology, #4511, 1:1000), anti-ERK (Cell Signaling Technology, #4695, 1:1000), anti-phospho-ERK (Cell Signaling Technology, #4370, 1:1000), anti-phospho-IKKβ (Cell Signaling Technology, #2078, 1:1000), anti-α-Tubulin (Sigma-Aldrich, T5168, 1:4000), anti-LaminB1 (Proteintech, 12987-1-AP, 1:1000) or anti-GAPDH (Millipore, USA, ABS16, 1:2000) antibody. Anti-rabbit or -mouse horseradish peroxidase-conjugated antibody was used as a secondary antibody, followed by enhanced chemiluminescence Western blotting detection reagents (Wako, Japan). Without LPS treatment, the expression level of cytokine was almost none, and in some cases, it was below the detection sensitivity. Therefore, we examined the time-course of target protein expression and compared at the point with the highest expression level of target protein. For quantification of expression level of target protein, the band intensities of scramble siRNA (control) and SNX25 siRNA-treated sample were measured and calculated the intensity ratio of SNX25 siRNA-sample to control. SNX25 siRNA-treated sample data were expressed as fold changes to control (scrambled siRNA) data and the same analysis was repeated for every three to seven independent experiments.

### Real-time quantitative RT-PCR

Total RNA was extracted using a NucleoSpin RNA kit (Macherey-Nagal, Germany) and reverse-transcribed using a QuantiTect Reverse Transcription kit (QIAGEN, Germany) with random primers. Real-time quantitative PCR was conducted in a Thermal Cycler Dice (Takara, Japan), using THUNDERBIRD SYBR qPCR Mix (Toyobo, Japan). PCR primers used in this study were β-actin primer (sense: AGCCATGTACGTAGCCATCC; antisense: CTCTCAGCTGTGGTGGTGAA), IL-1β primer (sense: CTCCATGAGCTTTGTACAAGG; antisense: TGCTGATGTACCAGTTGGGG), TNF-α primer (AATTCACTGGAGCCTCGAATG), IL-6 primer (sense: ACACATGTTCTCTGGGAAATCG; antisense: TGAAGGACTCTGGCTTTGTC) and SNX25 primer (sense: CATGGATCGTGTTCTGAGAG; antisense: GAAGTCATCTAAGAGCAGGATGG).

### Nuclear and cytoplasmic extraction

Nuclear and cytoplasmic extract was isolated from RAW 264.7 cell lysates using a nuclear extraction kit (abcam, ab113474) according to the manufacturer’s instructions.

### Statistical analysis

Quantitative analysis was performed from at least three independent experimental groups. All data are shown as mean ± SD. Statistical analysis was performed using Welch’s t test for two groups or Tukey–Kramer method for multiple groups. Statistical significance is indicated as asterisks. * *P* < 0.05, ** *P* < 0.01, *** *P* < 0.001. All n and *P* values are indicated in figure legends.

## Results

### SNX25 regulates LPS-induced proinflammatory cytokine expression

**Fig 1:**
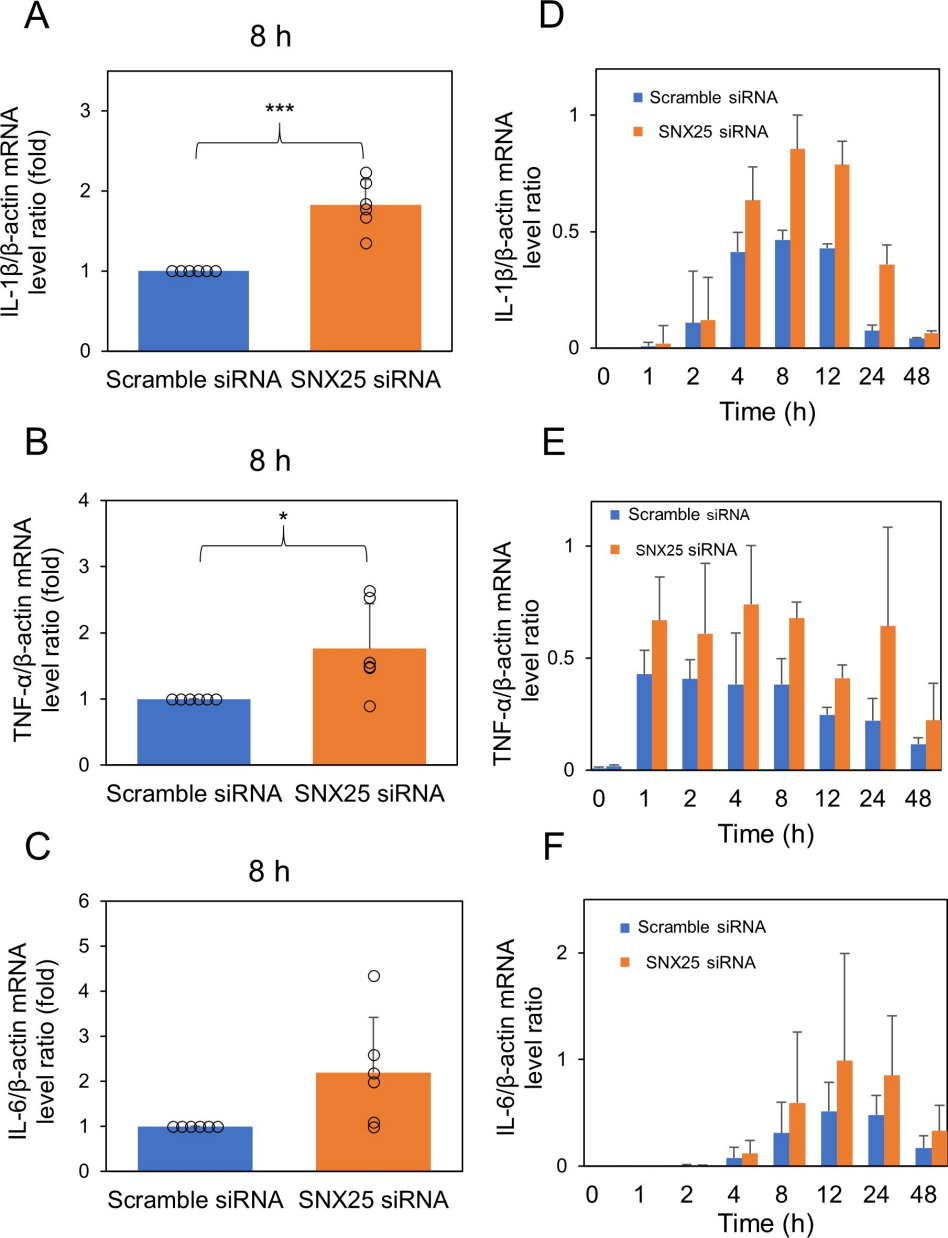
Relative mRNA expression of proinflammatory cytokines in SNX25 knockdown RAW 264.7 cells after LPS stimulation. (A–C) RAW 264.7 cells were stimulated with LPS (1μg/ml) for 8 h and mRNA was extracted immediately. RT-qPCR analyzed the relative expression of IL-1β, TNF-α, and IL-6. β-actin was used as an endogenous control. All data are expressed as means ± SD. Circles indicate the individual experimental values. * *P* < 0.05 *** *P* < 0.001, n = 6, Welch’s t-test. (D–F) RAW 264.7 cells were stimulated with LPS (1μg/ml) for various times and mRNA was extracted immediately. RT-qPCR analyzed the relative expression of IL-1β, TNF-α, and IL-6. β-actin was used as an endogenous control. All data are expressed as means ± SD. n = 3.

**Fig 2:**
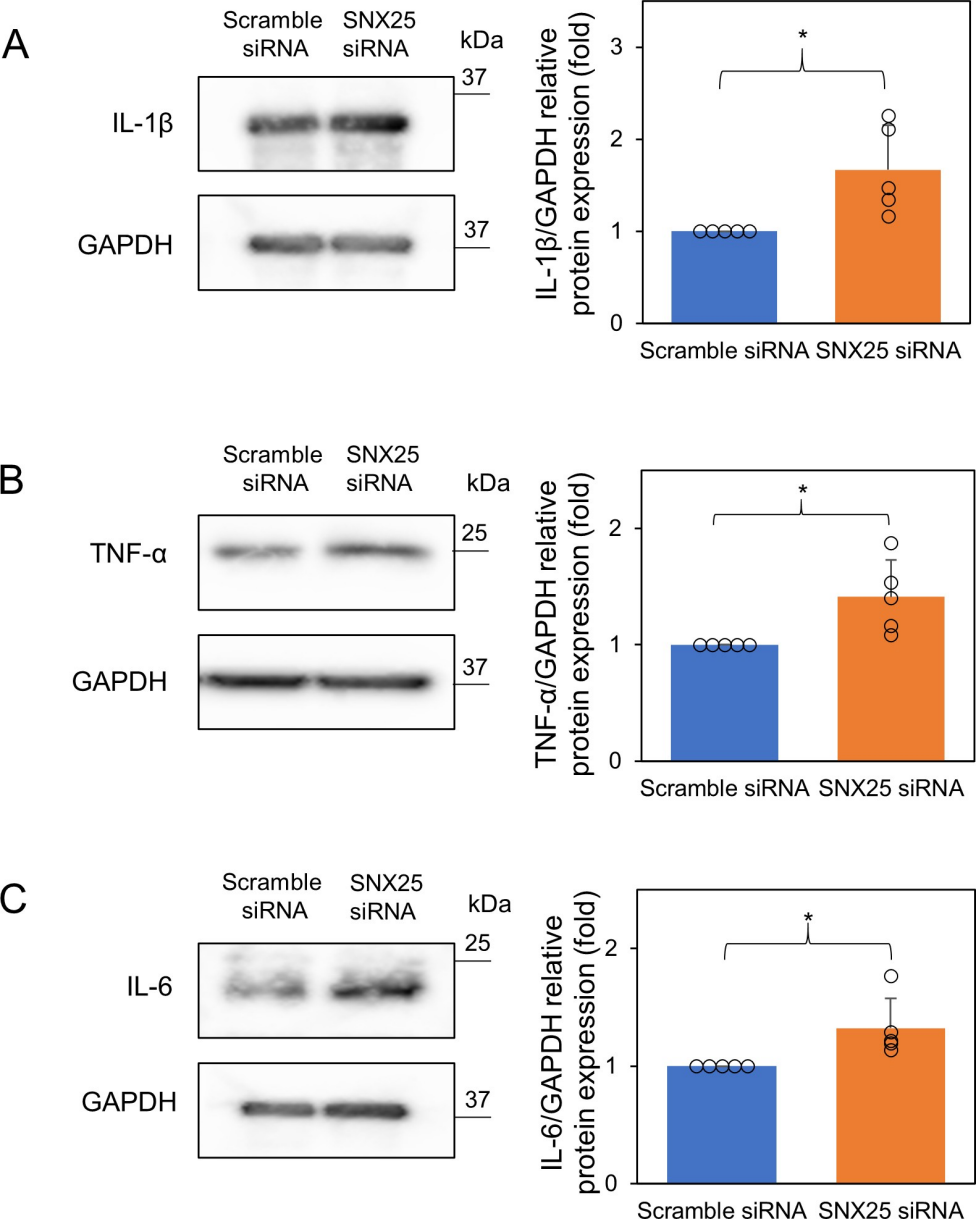
Relative protein expression of proinflammatory cytokines in SNX25 knockdown RAW 264.7 cells after LPS stimulation. (A–C) RAW 264.7 cells were stimulated with LPS (1μg/ml) for 12 h. The expression of IL-1β, TNF-α, and IL-6 was analyzed by Western blotting. GAPDH was used as a loading control. Data are expressed as means ± SD. Circles indicate the individual experimental values. * *P* < 0.05, n = 5.

First, we knocked down SNX25 using two different SNX25 siRNAs (SNX25 siRNA-1 and SNX25 siRNA-2). SNX25 siRNA-2 decreased the SNX25 mRNA level more effectively (SNX25 siRNA-1, 0.77 ± 0.10-fold control; SNX25 siRNA-2, 0.49 ± 0.0075-fold control) (S1A Fig). SNX25 siRNA-2 also decreased the SNX25 protein level (S1B Fig). We therefore employed SNX25 siRNA-2 to knock down SNX25 hereafter. To investigate a role of SNX25 in inflammation, we detected proinflammatory cytokines in SNX25-knockdown RAW 264.7 cells after LPS stimulation. We examined the expression of IL-1β, TNF-α and IL-6 mRNAs using RT-qPCR and found that these cytokine mRNAs were more highly expressed at 8 h after LPS treatment in RAW 264.7 transfected with SNX25 siRNA (IL-1β, SNX25 siRNA, 1.8 ± 0.32-fold control *P* = 0.0014; TNF-α, SNX25 siRNA, 1.8 ± 0.68-fold control *P* = 0.041; IL-6, SNX25 siRNA, 2.2 ± 1.2-fold control *P* = 0.063) (Fig 1A–1C). We also measured the time courses of these cytokine mRNA levels after LPS stimulation. We detected no marked difference in time to peak between control cells and SNX25 knockdown cells (Fig 1D–1F). SNX25 knockdown affects the expression levels of inflammatory cytokines but not the time of maximal expression after LPS treatment. We next examined whether SNX25 knockdown changes protein levels of these cytokines by Western blotting. IL-1β, TNF-α and IL-6 levels also increased at 12 h after LPS treatment (IL-1β, SNX25 siRNA, 1.7 ± 0.48-fold control *P* = 0.037; TNF-α, SNX25 siRNA, 1.4 ± 0.32-fold control *P* = 0.019; IL-6, SNX25 siRNA, 1.3 ± 0.26-fold control *P* = 0.048) (Fig 2A–2C). These results suggest that SNX25 negatively regulates proinflammatory cytokine expression in macrophages.

### SNX25 inhibits NF-κB translocation into the nucleus

**Fig 3:**
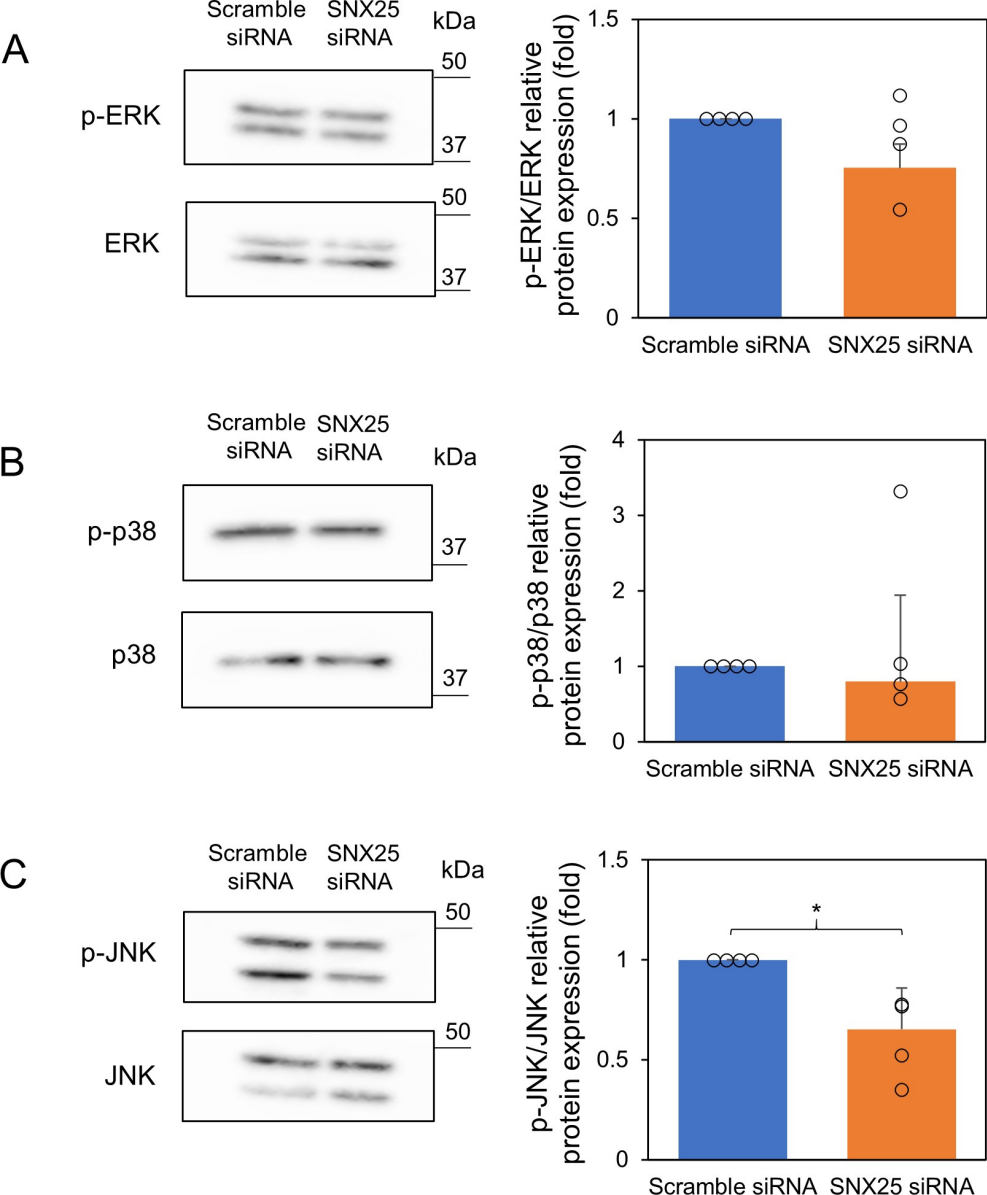
Activation of MAPK signaling pathways in SNX25 knockdown RAW 264.7 cells. (A–C) RAW 264.7 cells were stimulated with LPS (1μg/ml) for 20 min. The expression of p-ERK, p-p38 and p-JNK was analyzed by Western blotting. ERK, p38, and JNK were used as loading controls. Data are expressed as means ± SD. Circles indicate the individual experimental values. * *P* < 0.05, n = 4.

MAPK signals (ERK, p38, and JNK) and NF-κB signal are important factors in proinflammatory cytokine expression. We therefore examined whether SNX25 knockdown affects MAPK phosphorylation at 20 min after LPS stimulation because all MAPK phosphorylation reached a peak at 20 min after LPS stimulation (S2A Fig). ERK and p38 phosphorylation were unchanged by SNX25 knockdown (p-ERK, SNX25 siRNA, 0.75 ± 0.12-fold control; p-p38, SNX25 siRNA, 0.80 ± 1.1-fold control) (Fig 3A and 3B). On the other hand, the level of JNK phosphorylation was decreased, after LPS stimulation, by SNX25 knockdown (SNX25 siRNA, 0.65 ± 0.21-fold control *P* = 0.032) (Fig 3C). This result contradicts the cytokine expression patterns (Figs 1 and 2); inhibition of JNK signaling negatively regulates cytokines and there must be a strong enhancer(s) that is activated by SNX25 knockdown to override JNK inhibition.

**Fig 4:**
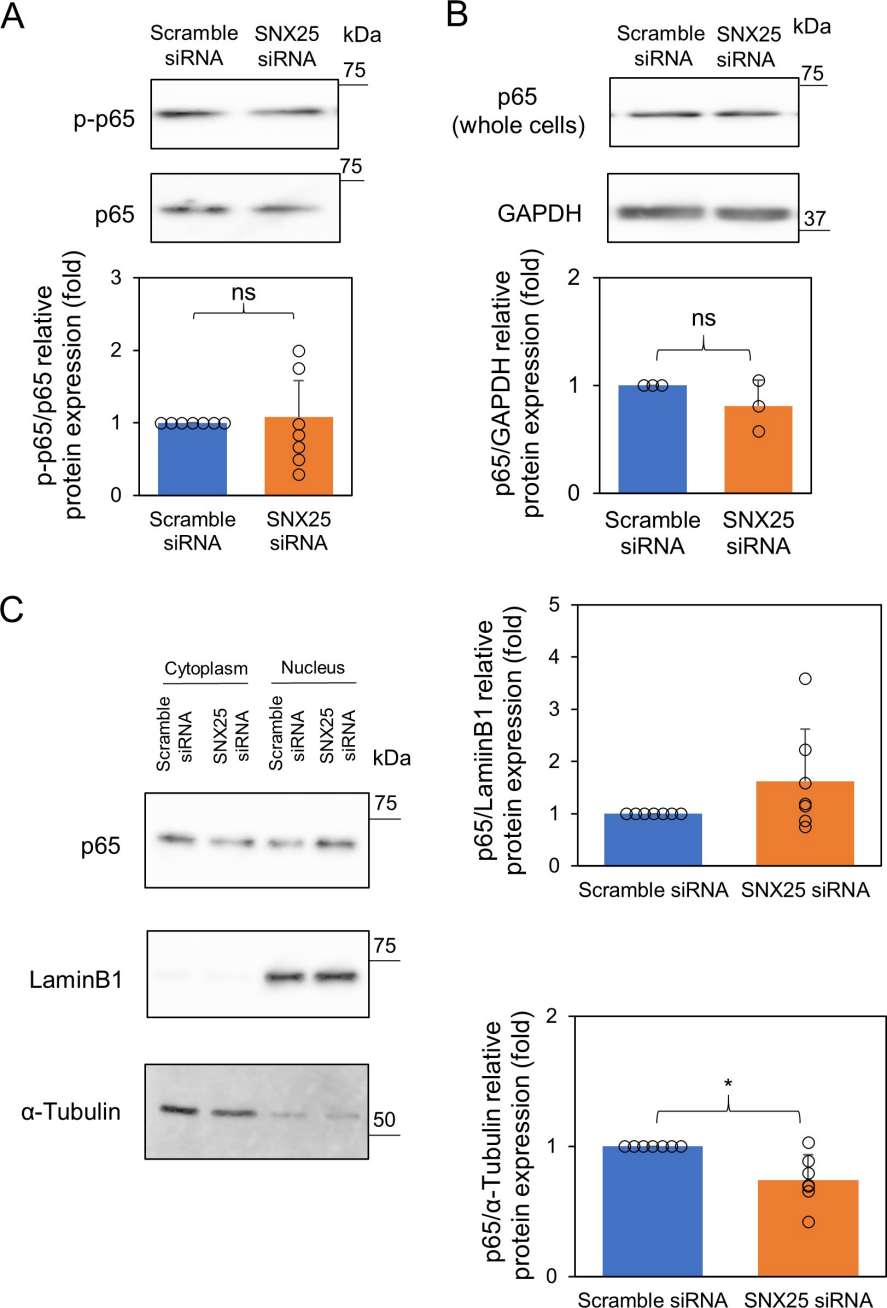
Activation of NF-κB signaling pathways in SNX25 knockdown RAW 264.7 cells. (A) RAW 264.7 cells were stimulated with LPS (1μg/ml) for 10 min. p-p65 expression was analyzed by Western blotting. p65 was used as a loading control. Circles indicate the individual experimental values. Data are expressed as means ± SD. n = 7. (B, C) RAW 264.7 cells were stimulated with LPS (1μg/ml) for 30 min. The expression of p65 in whole cells, cytoplasm and nucleus was analyzed by Western blotting. GAPDH, α-Tubulin and LaminB1 were used as a loading control. Data are expressed as means ± SD. Circles indicate the individual experimental values. * *P* < 0.01; ns, not significant, n = 3 (whole cells), n = 7 (nucleus and cytoplasm).

We hypothesized that the NF-κB signaling pathway is a candidate for such a strong enhancer. We first examined phosphorylation of p65 (at serine 536), which is a hallmark of NF-κB activation [18]. p65 phosphorylation reached a peak at 10 min after LPS stimulation (S2B Fig). There was no obvious difference in the level of phosphorylated p65 between SNX25 siRNA-treated cells and scramble siRNA-treated cells after 10 min LPS stimulation (SNX25 siRNA, 1.00 ± 0.64-fold control) (Fig 4A). We next asked whether SNX25 knockdown affects nuclear translocation of NF-κB. We confirmed the expression levels of p65 in whole-cell lysate are comparable in SNX25 siRNA-treated cells and scramble siRNA-treated cells (SNX25 siRNA, 0.81 ± 0.28-fold control) (Fig 4B). We extracted nuclear and cytoplasmic fraction from whole cell lysates to examine the expression of p65 in nucleus and cytoplasm. Nuclear translocation of p65 reached a peak at 30 min after LPS stimulation (S2C Fig). SNX25 knockdown decreased cytoplasmic p65 after 30 min LPS stimulation (SNX25 siRNA, 0.74 ± 0.19-fold control *P* = 0.012). In addition, SNX25 knockdown increased nuclear p65 though the difference was not statistically significant (SNX25 siRNA, 1.50 ± 0.84-fold control *P* = 0.15) (Fig 4C). These results suggest that SNX25 knockdown increases translocation of p65 to the nucleus and thereby enhances cytokine expression.

### SNX25 suppresses ubiquitination of IκBα

**Fig 5:**
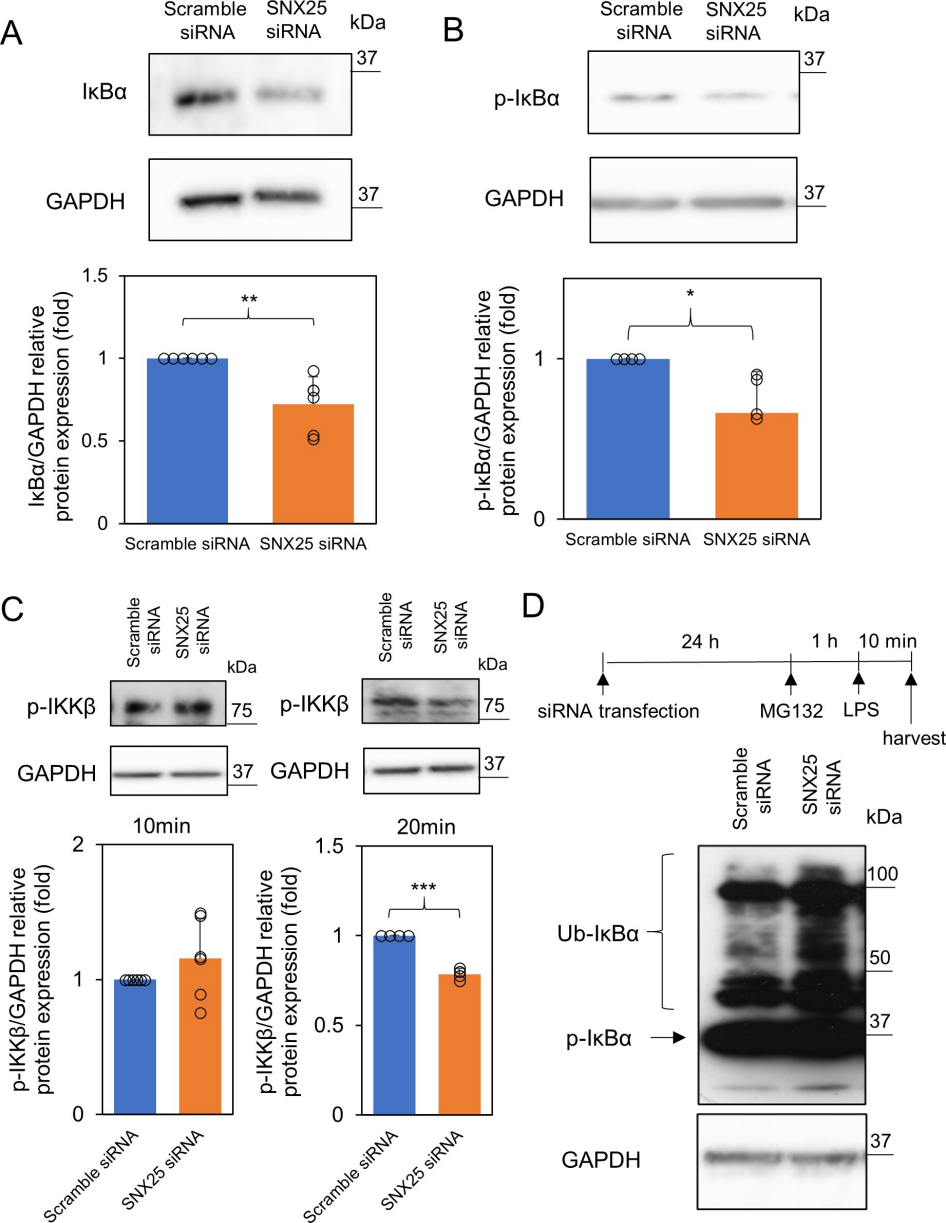
Activation of IκBα and IKKβ in SNX25 knockdown RAW 264.7 cells. (A and B) RAW 264.7 cells were stimulated with LPS (1μg/ml). Expression of IκBα and p-IκBα was analyzed by Western blotting. GAPDH was used as a loading control. Data are expressed as means ± SD. Circles indicate the individual experimental values. * *P* < 0.05 ** *P* < 0.01, n = 4 (p-IκBα) n = 6 (IκBα). (C) RAW 264.7 cells were stimulated with LPS (1μg/ml). Expression of p-IKKβ was analyzed by Western blotting. GAPDH was used as a loading control. Data are expressed as means ± SD. Circles indicate the individual experimental values. *** *P* < 0.001, n = 4. (D) MG132-induced accumulation of polyubiquitinated IκBα was analyzed by Western blotting. GAPDH was used as a loading control.

We next focused on the mechanisms underlying NF-κB nuclear translocation. Since nuclear translocation of NF-κB depends on ubiquitin-mediated proteasomal degradation of IκBα [19], we examined the degradation of IκBα after LPS stimulation. IκBα degradation reached a peak at 20 min after LPS stimulation (S2D Fig). SNX25 knockdown promoted IκBα degradation after 20 min LPS stimulation (SNX25 siRNA, 0.72 ± 0.16-fold control *P* = 0.009) (Fig 5A). IκBα is phosphorylated by p-IKKβ and is degraded by the ubiquitin-proteasome system [4]. Therefore, we hypothesized that SNX25 knockdown would promote phosphorylation of IκBα by p-IKKβ. Contrary to this expectation, p-IκBα expression decreased after 10 min LPS stimulation (p-IkBa, SNX25 siRNA, 0.76 ± 0.14-fold control *P* = 0.046) (Fig 5B). The expression of p-IKKβ also decreased after 20 min LPS stimulation (p-IKKβ, SNX25 siRNA, 0.76 ± 0.050-fold control *P* = 0.00080) though there was no obvious difference in the expression of p-IKKβ at the peak time, 10min after LPS stimulation (Fig 5C). As an alternative route for IκBα degradation, ubiquitination of IκBα might be promoted by SNX25 knockdown. To test this possibility, SNX25 knockdown cells and control cells were treated with the proteasome inhibitor MG132 (5 μM, 1 h) and then subjected to Western blotting with p-IκBα antibody. An increased level of poly-ubiquitinated IκBα was observed in SNX25 knockdown cells compared with control cells after 10 min LPS stimulation (Fig 5D, Ub-IκBα). These results suggest that SNX25 negatively regulates ubiquitin-mediated IκBα degradation.

## Discussion

Sorting nexins play key roles in membrane trafficking, membrane remodeling, and organelle motility [10] and some of them are involved in the immune system [11–13]. SNX25 is reported to negatively regulate TGF-β signaling by enhancing degradation of the receptor TGFbRI [14]. TGF-β signaling is one of the key factors in the immune system, and SNX25 may therefore be involved in immunity. However, its precise functions remain to be determined. For the first time, we found that SNX25 is an important regulator of the TLR4 signaling pathway.

We focused on innate immunity using the macrophage cell line RAW 264.7. To examine whether SNX25 affects innate immunity, we examined proinflammatory cytokine expression in SNX25 knockdown RAW 264.7 cells treated with LPS. We found that SNX25 knockdown increased the expression of IL-1β, TNF-α, and IL-6 (Figs 1A–1C and 2). We also found that SNX25 did not affect the time-course profiles of these cytokines’ mRNA levels after LPS stimulation (Fig 1D–1F), indicating that SNX25 suppresses LPS-induced proinflammatory cytokine expression without changing its kinetics.

The MAPK pathway has an important role in cytokine expression [20]; however, contrary to our expectation, SNX25 knockdown did not activate MAPK (Fig 3). We therefore focused on the NF-κB pathway as a target for inflammation regulation by SNX25. The NF-κB pathway is also an important factor in the expression of proinflammatory cytokines [21]. We found that SNX25 knockdown promotes nuclear translocation of NF-κB (Fig 4). This result indicates that SNX25 regulates proinflammatory cytokines via the NF-κB signal. In the canonical NF-κB pathway, IκBα is phosphorylated by p-IKKβ and then degraded by the ubiquitin-proteasome system [4]. We found that SNX25 knockdown promotes IκBα ubiquitination and degradation (Fig 5). These results indicate that SNX25 inhibits ubiquitination of IκBα. SNX25 is probably a powerful suppressor of ubiquitination of IκBα because its knockdown increased the expression of polyubiquitinated IκBα despite inhibition of IKKβ, upstream of IκBα. The effect of SNX25 on IκBα ubiquitination can be explained by several possible mechanisms such as the inhibition of SCF^β-TrCP^, a specific E3 ubiquitin ligase of IκBα, and activation of deubiquitinating enzymes (DUBs) against IκBα [5]. Another SNX family member, SNX17, was reported by Pengtao et al. to recruit the DUB USP9X to antagonize mindbomb1-mediated ubiquitination and degradation of PCM1, one of the centriolar satellite proteins, during serum starvation-induced ciliogenesis [22]. Their conclusion and our results raise the possibility that SNX25 recruits a DUB against IκBα, leading to inhibition of IκBα ubiquitination.

Besides SNX17, other SNXs are also reported to be associated with the ubiquitin-proteasome pathway. For example, SNX16 activates c-Myc signaling by inhibiting ubiquitin-mediated proteasomal degradation of eukaryotic translation elongation factor 1A2 in colorectal cancer development [23]. This study revealed that SNX16 antagonizes eukaryotic translation elongation factor 1A2 ubiquitination and activates c-Myc signaling, thereby driving tumorigenesis. SNX5 enhances K48 ubiquitination of retinoic acid-inducible gene I (RIG-I) and attenuates K63 ubiquitination of RIG-I, resulting in inhibition of the RIG-I-mediated innate immune response [24]. These reports and the present study indicate that several SNX family members play an important role in regulation of the ubiquitin-proteasome pathway. Some of the SNX family functions can be attributed to protein ubiquitination or de-ubiquitination. For example, recent studies showed that ubiquitination levels are involved in receptor endocytosis, one of the main roles of the SNX family [25, 26]. Further research about the relationship between SNXs and protein ubiquitination will reveal the overall function of the SNX family.

We also found that SNX25 knockdown decreases phosphorylation of IKKβ. This indicates that SNX25 knockdown suppresses a molecule(s) upstream of IKKβ. In addition, we found SNX25 knockdown did not affect the expression of TLR4 (data not shown). SNX25 is reported to downregulate TGFβ signaling. Therefore, these results suggest that TGFβ signaling is activated by SNX25 knockdown, resulting in downregulation of TLR4 signaling because TGFβ signaling inhibits TLR4 signaling [27]. On the other hand, some known factors upstream of IKKβ undergo ubiquitin-mediated degradation. For example, TRAF6 is reportedly inhibited by ubiquitin-mediated degradation [28]. SNX25 may also affect not only IκBα but also upstream factors such as TRAF6 via ubiquitin-mediated degradation. Thus, further experiments are needed to determine the entire molecular mechanism of SNX25 against IKKβ.

**Fig 6:**
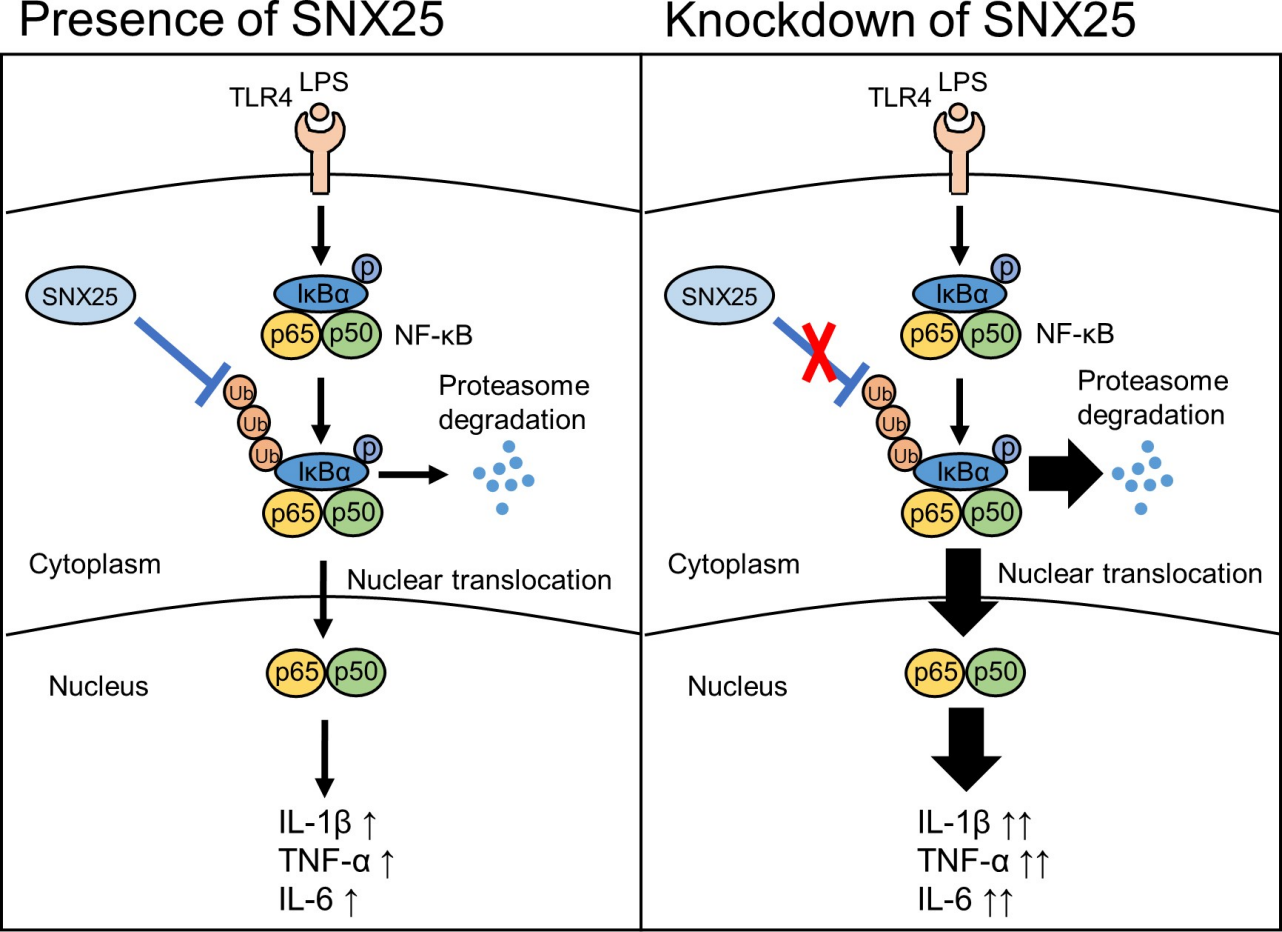
Model illustrating that SNX25 regulates proinflammatory cytokine expression by affecting IκBα ubiquitination. SNX25 inhibits ubiquitination of IκBα and the dissociation of IκBα/NF-κB (p65 and p50) complex and prevents nuclear translocation of NF-κB and subsequent cytokine expression.

In conclusion, we showed several functional roles of SNX25 in TLR4 signaling such as ubiquitination of IκBα and the expression of inflammatory cytokines (Fig 6). Further investigation of the mechanism of IκBα ubiquitination by SNX25 should provide new insights into the crucial role of this SNX under inflammatory conditions.

In the case of SNX25-knockdowned cell, ubiquitination and proteasome degradation of IκBα are enhanced. p65 and p50, released from the complex with IκBα, translocate into nucleus and activate cytokine transcription and expression.

## Supporting information

S1 Fig Relative SNX25 levels in SNX25 knockdown RAW 264.7 cells using each siRNA. (A) SNX25 siRNA-1 and siRNA-2 were transfected into RAW 264.7 cells. RT-qPCR analyzed the relative mRNA expression of SNX25. β-actin was used as endogenous control. Data are expressed as means ± SD. n = 3 ** P<0.01, Tukey–Kramer method. (B) SNX25 siRNA-2 was transfected into RAW 264.7 cells. RAW 264.7 cells were incubated for 24h or 48h. The protein level of SNX25 was analyzed by Western blotting. GAPDH was used as a loading control.
(TIF)

S2 Fig LPS time-course of inflammation-related factors. (A-D) RAW 264.7 cells were stimulated with LPS (1μg/ml) for 10min, 20min, 30min or 1h. The expression of p-ERK, p-p38, p-JNK, IκBα, p65, p-IκBα, p-p65, p-IKKβ was analyzed by Western blotting. ERK, p-38, JNK, GAPDH, LaminB1 and α-Tubulin was used as a loading control.
(TIF)

S1 Raw images (PDF)
