# Supplementary figures and images for: SNX25 regulates proinflammatory cytokine expression via the NF-κB signal in macrophages
Source: PLoS One. 2021 Mar 1;16(3):e0247840. doi: 10.1371/journal.pone.0247840 (PMC7920363; doi:10.1371/journal.pone.0247840)

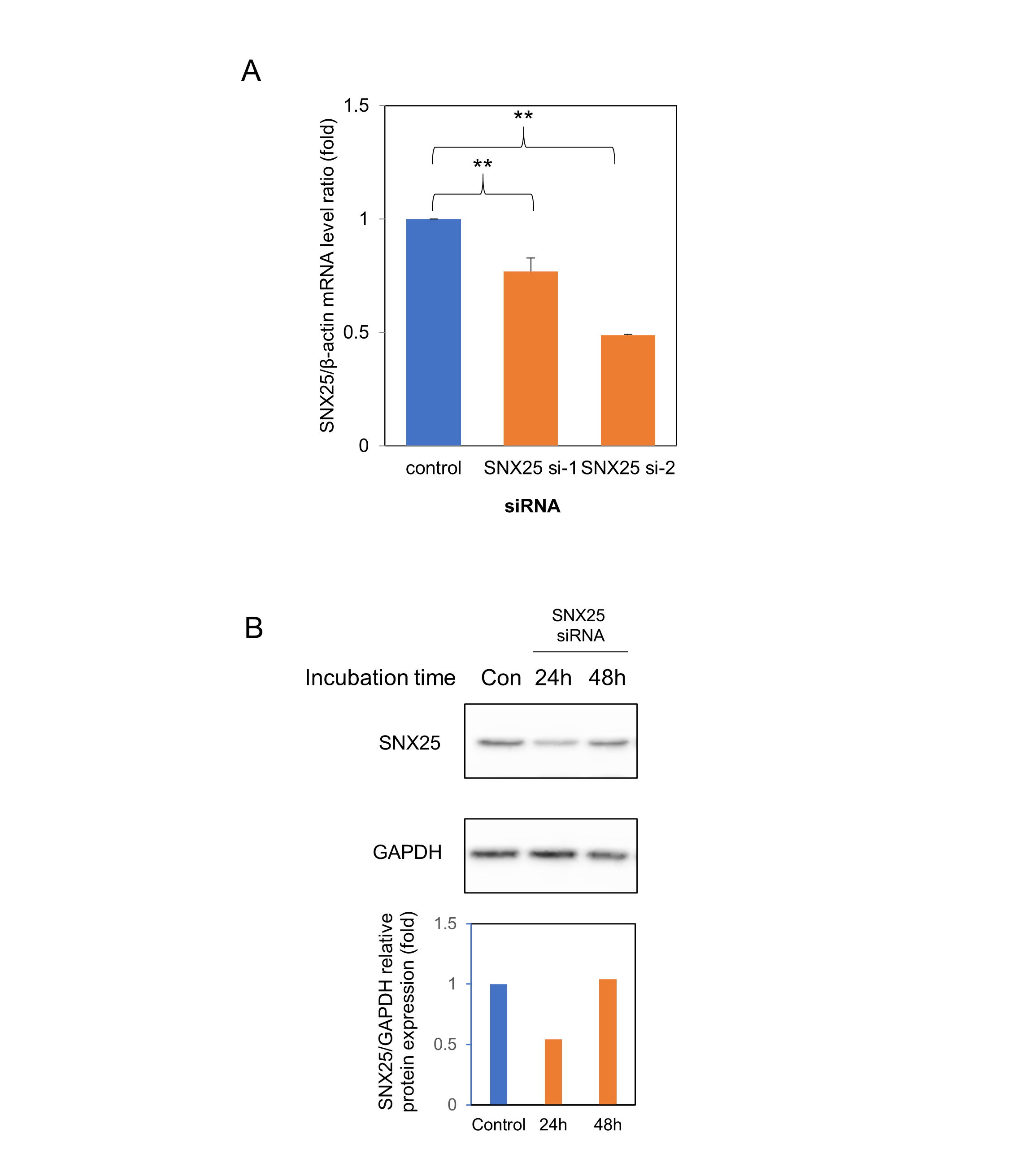

Supplement: S1 Fig — (A) SNX25 siRNA-1 and siRNA-2 were transfected into RAW 264.7 cells. RT-qPCR analyzed the relative mRNA expression of SNX25. β-actin was used as endogenous control. Data are expressed as means ± SD. n = 3 ** P<0.01, Tukey–Kramer method. (B) SNX25 siRNA-2 was transfected into RAW 264.7 cells. RAW 264.7 cells were incubated for 24h or 48h. The protein level of SNX25 was analyzed by Western blotting. GAPDH was used as a loading control. (TIF) [file pone.0247840.s001.tif]

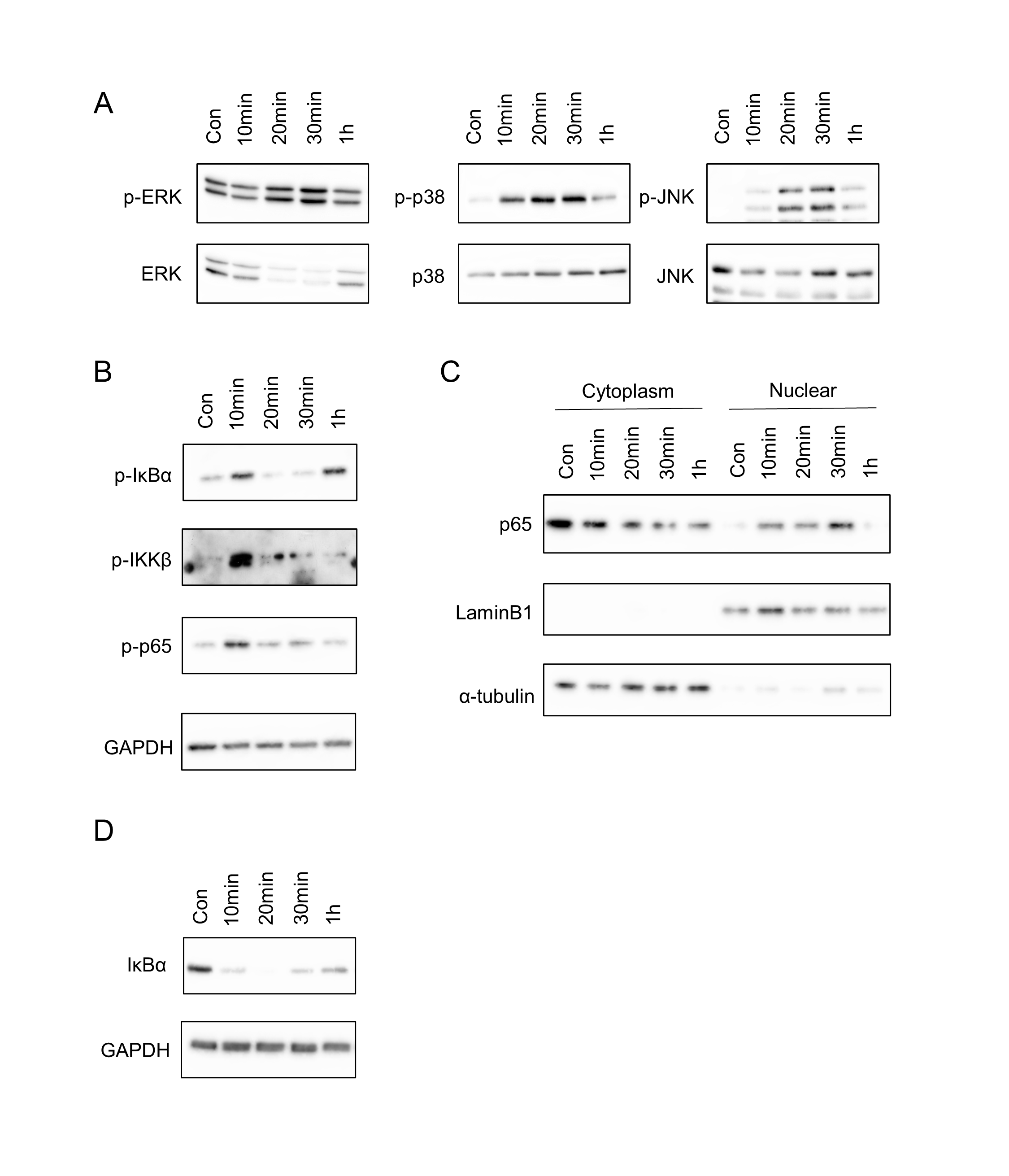

Supplement: S2 Fig — (A-D) RAW 264.7 cells were stimulated with LPS (1μg/ml) for 10min, 20min, 30min or 1h. The expression of p-ERK, p-p38, p-JNK, IκBα, p65, p-IκBα, p-p65, p-IKKβ was analyzed by Western blotting. ERK, p-38, JNK, GAPDH, LaminB1 and α-Tubulin was used as a loading control. (TIF) [file pone.0247840.s002.tif]

Fig 2

A

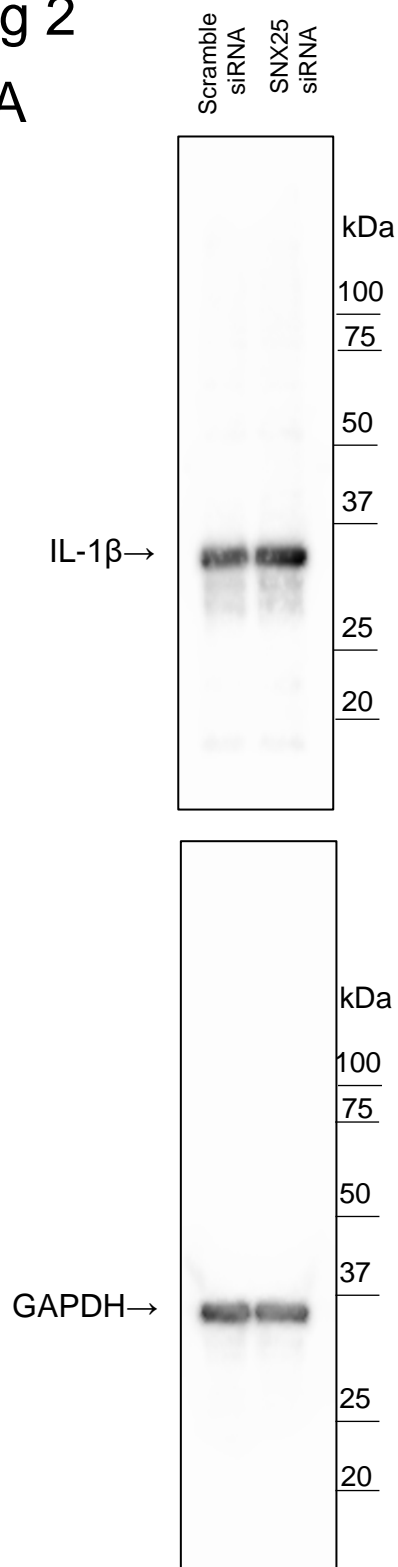

B

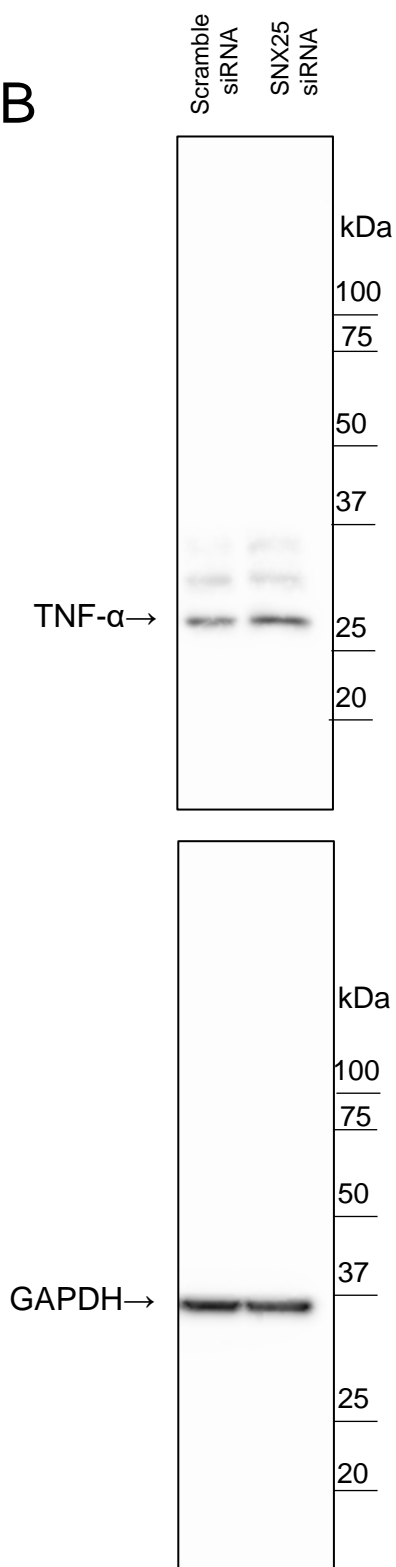

C

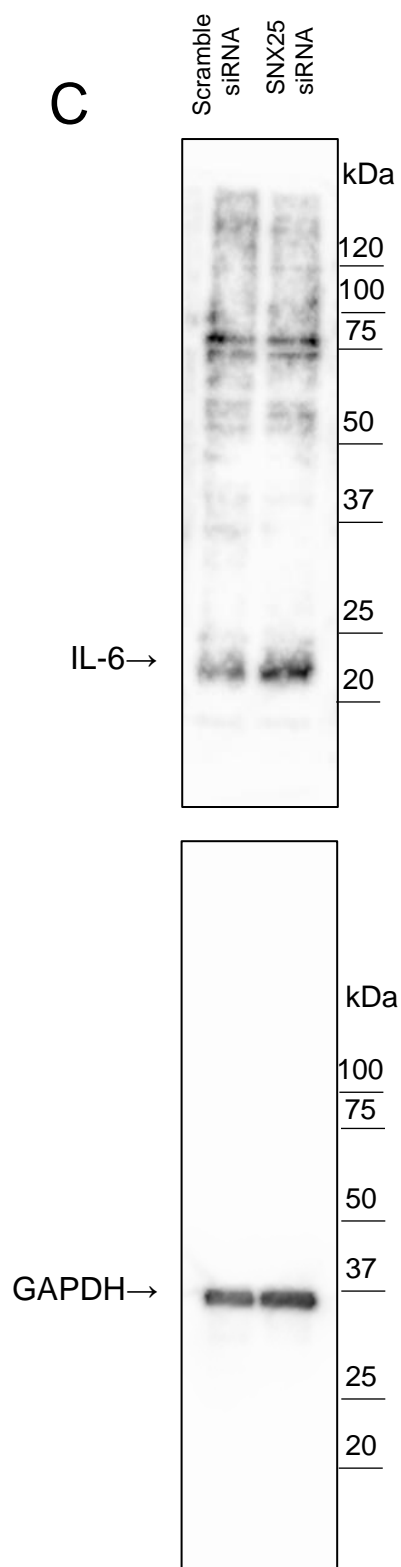

Fig 3

A

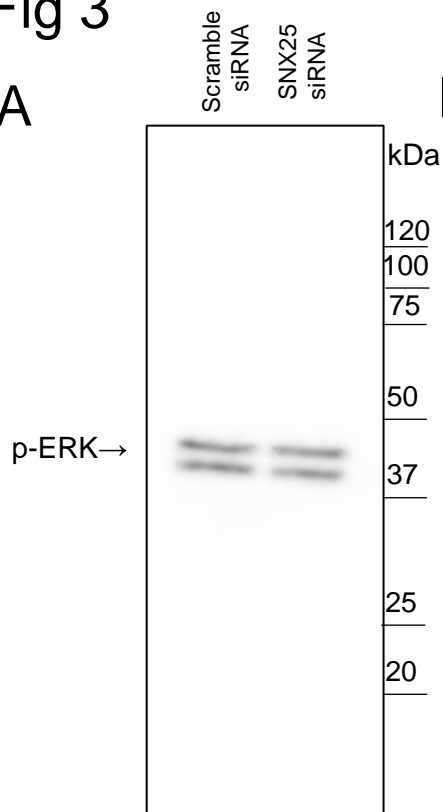

B

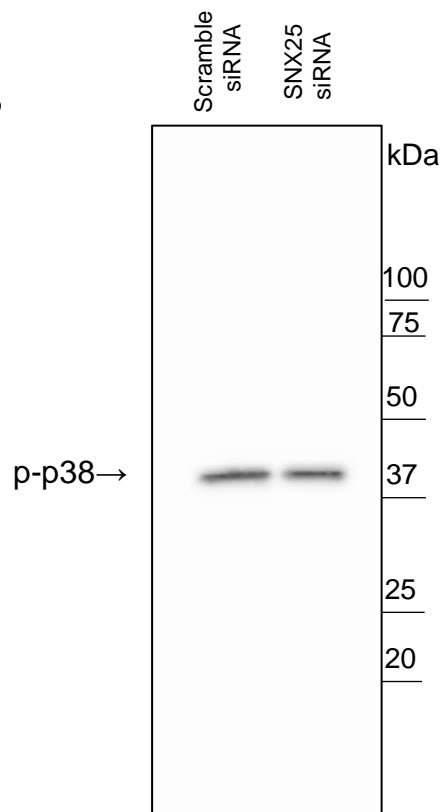

C

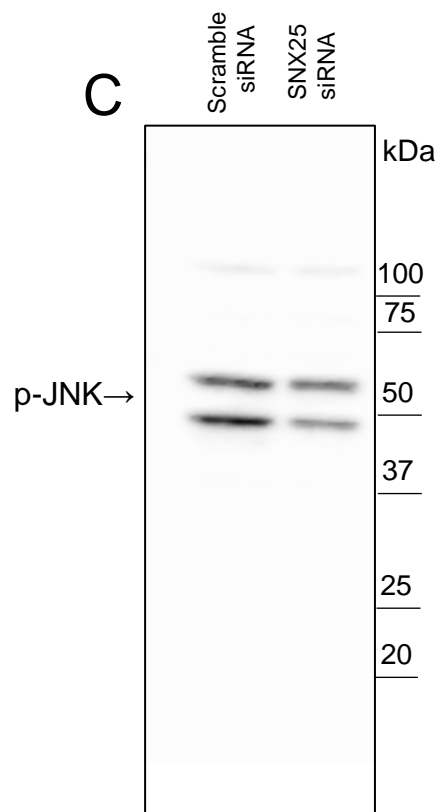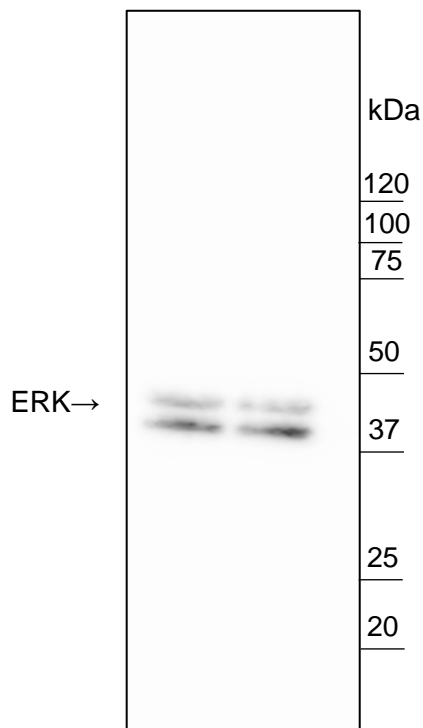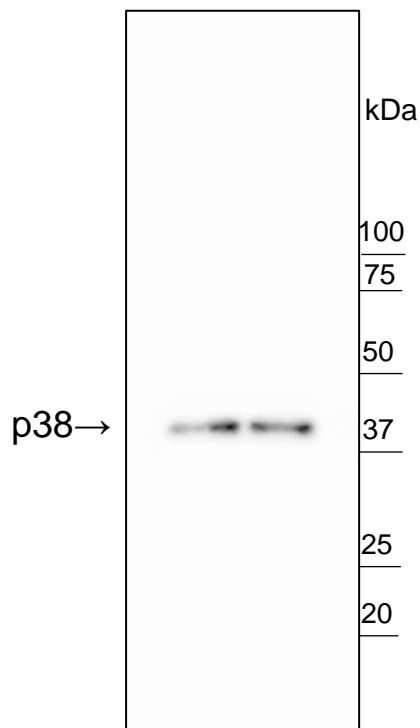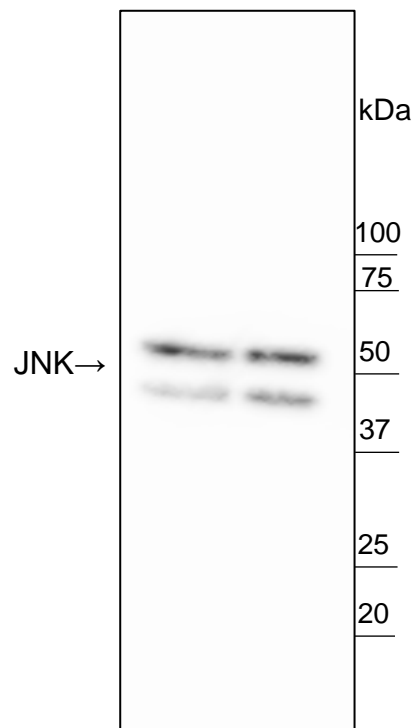

Fig 4

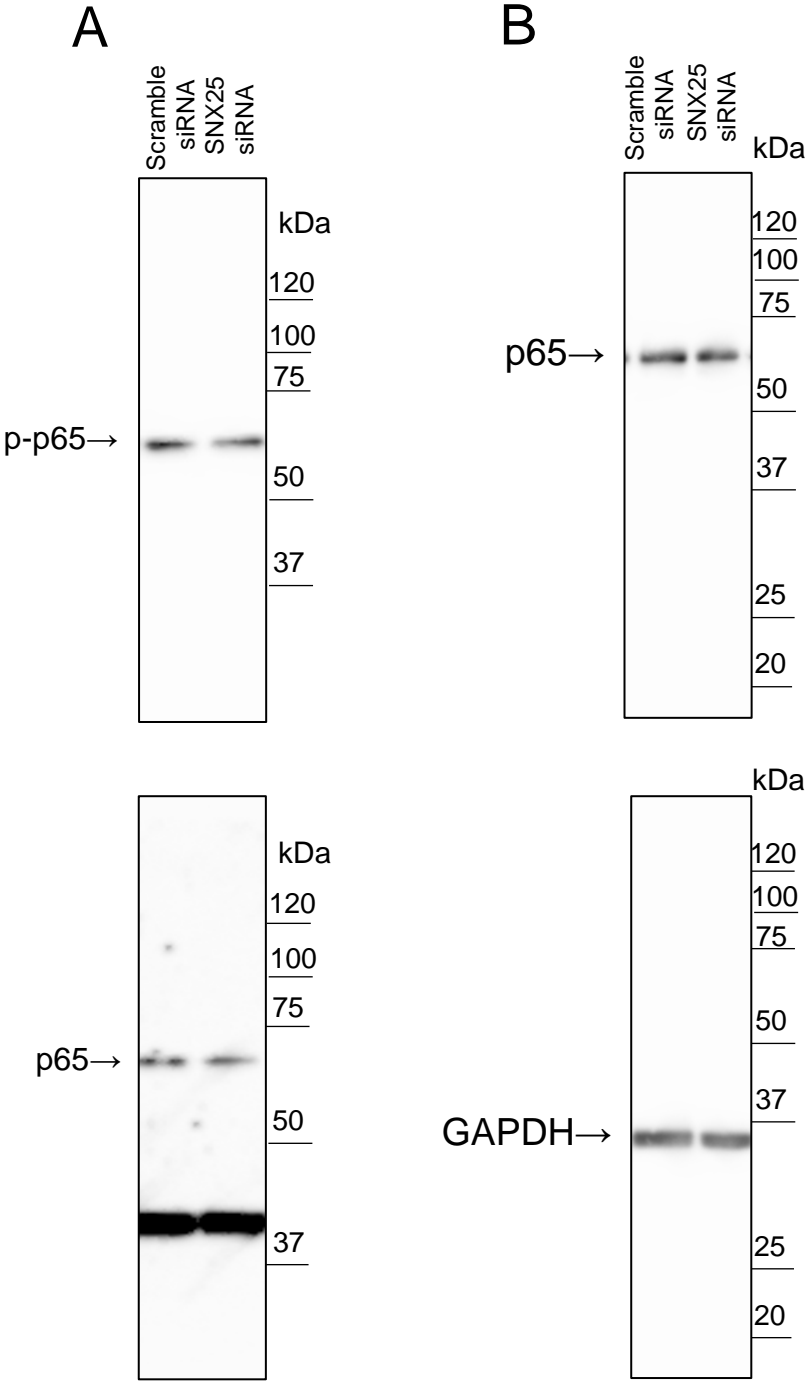

Fig 4

C

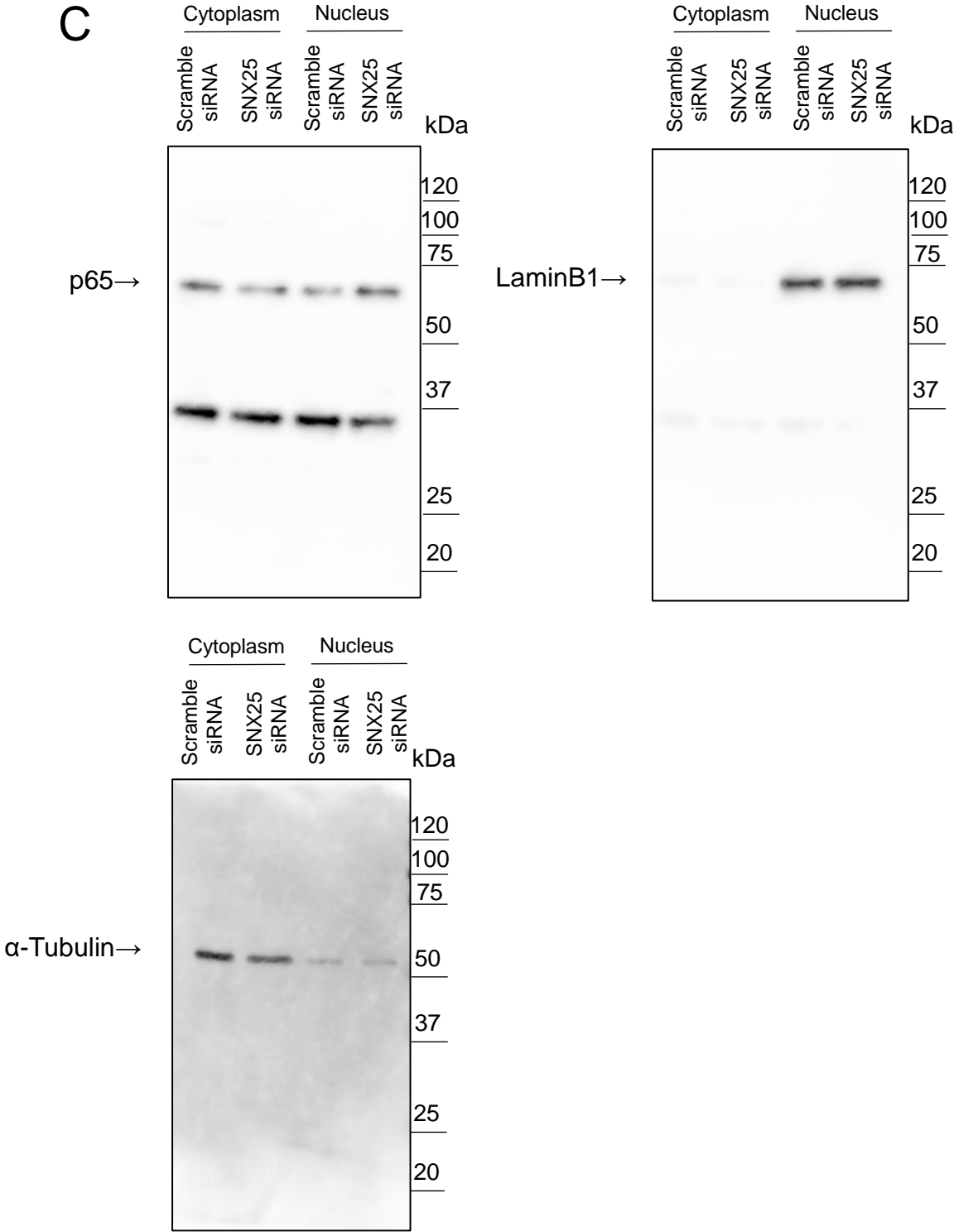

Fig 5

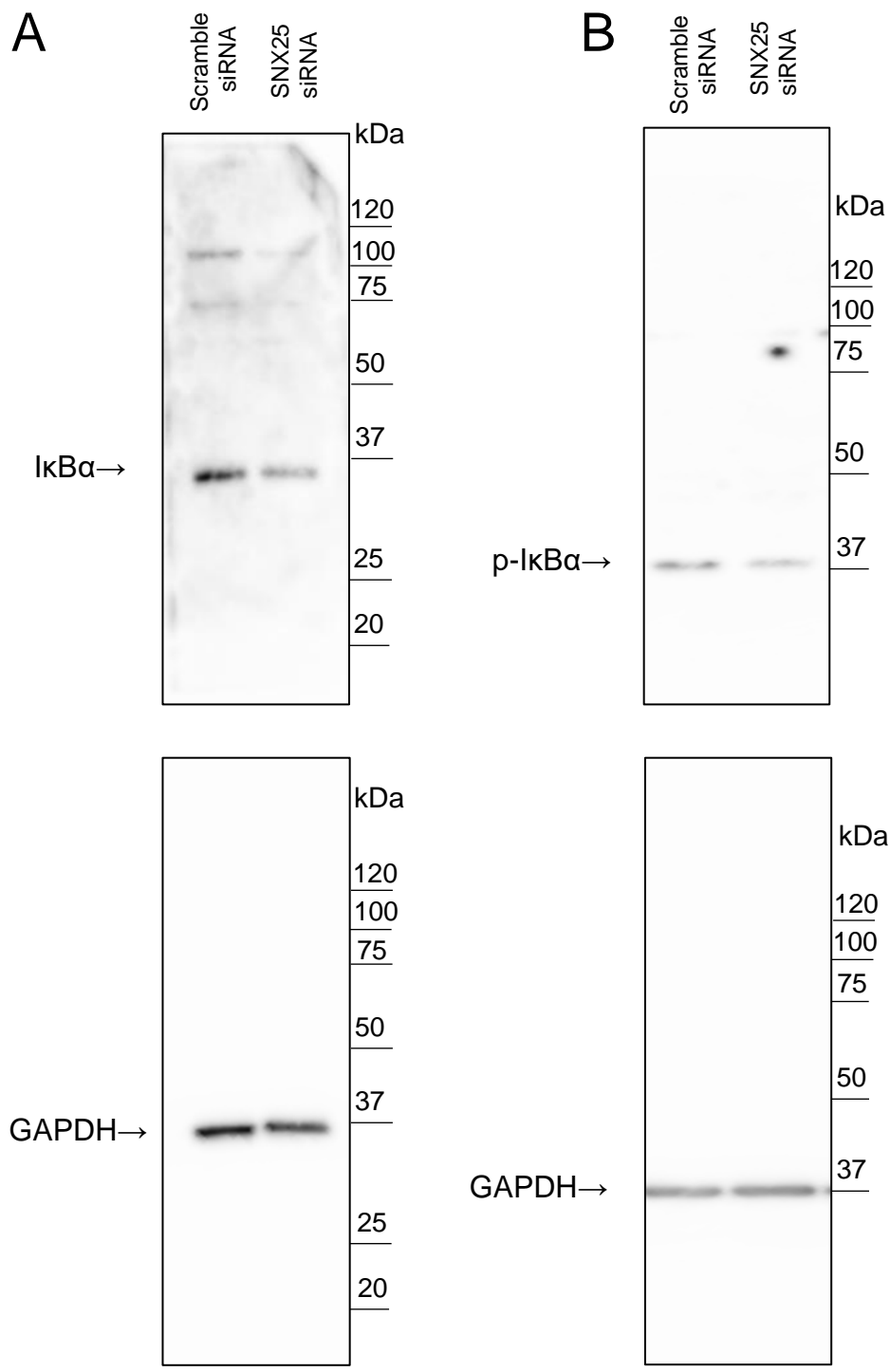

Fig 5

C

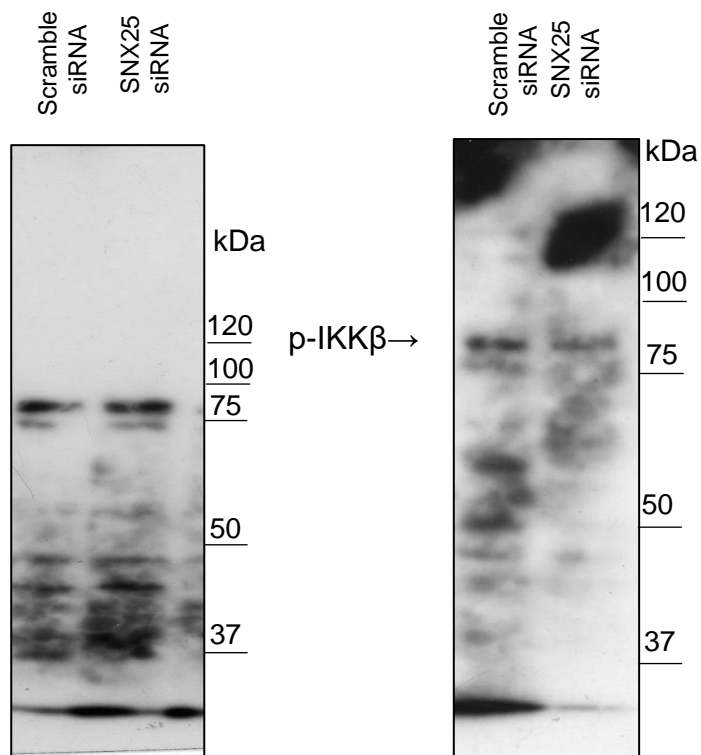

D

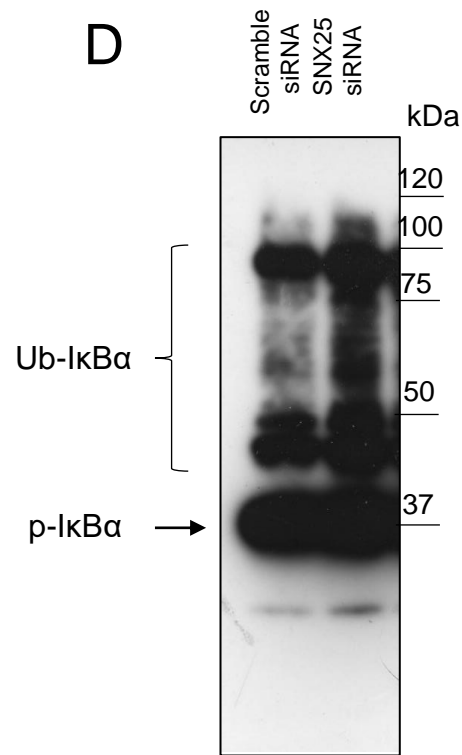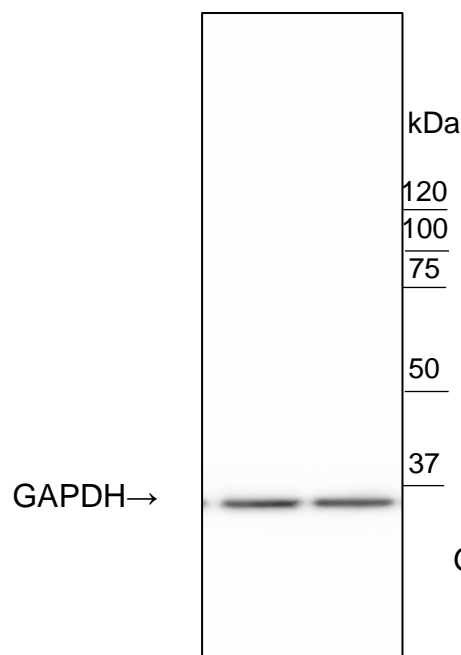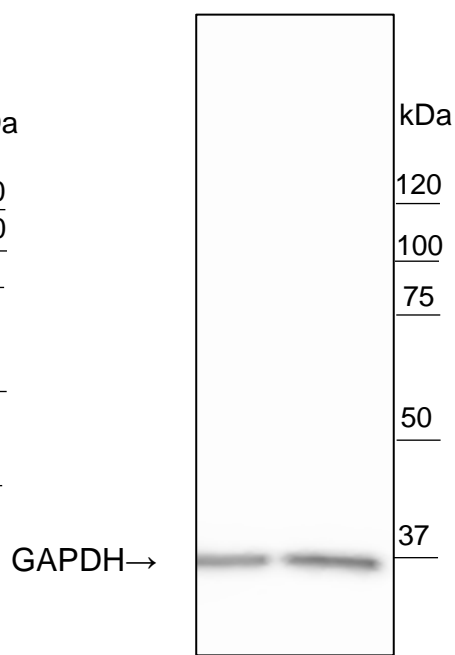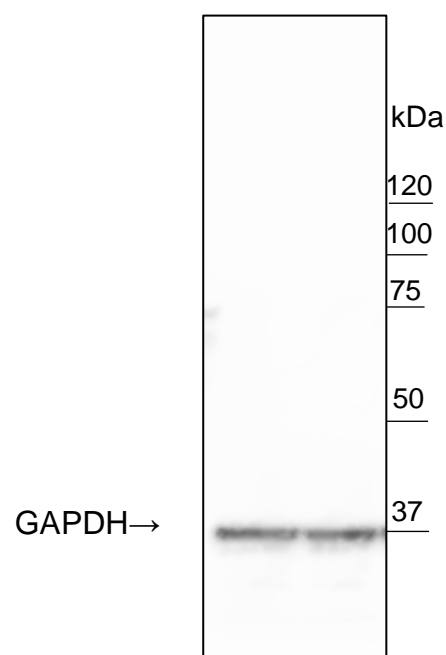

S1 Fig

B

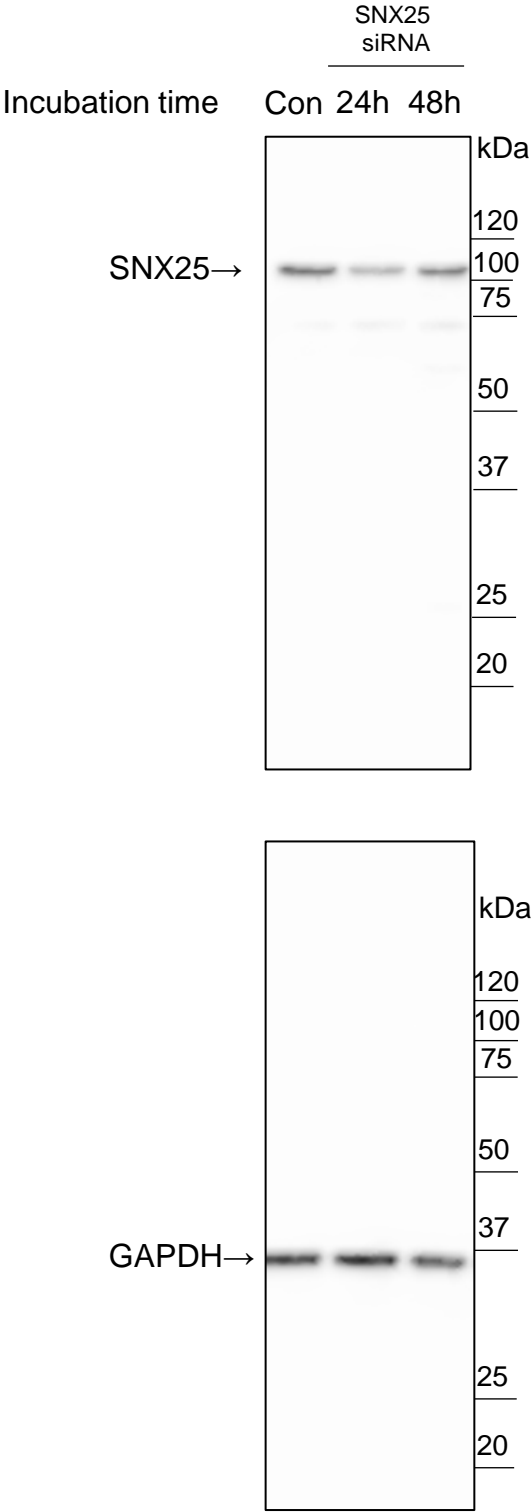

S2 Fig  
A

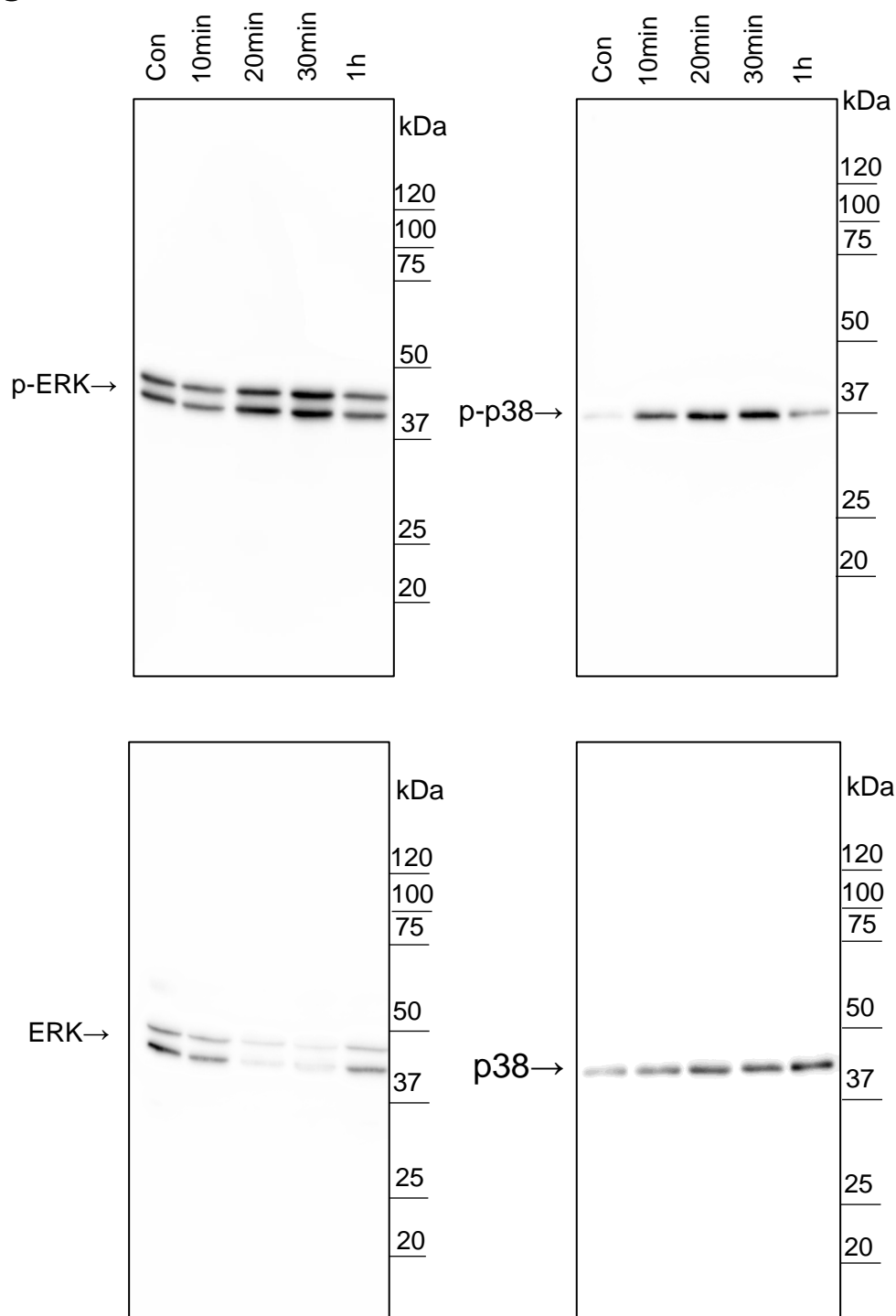

S2 Fig  
A

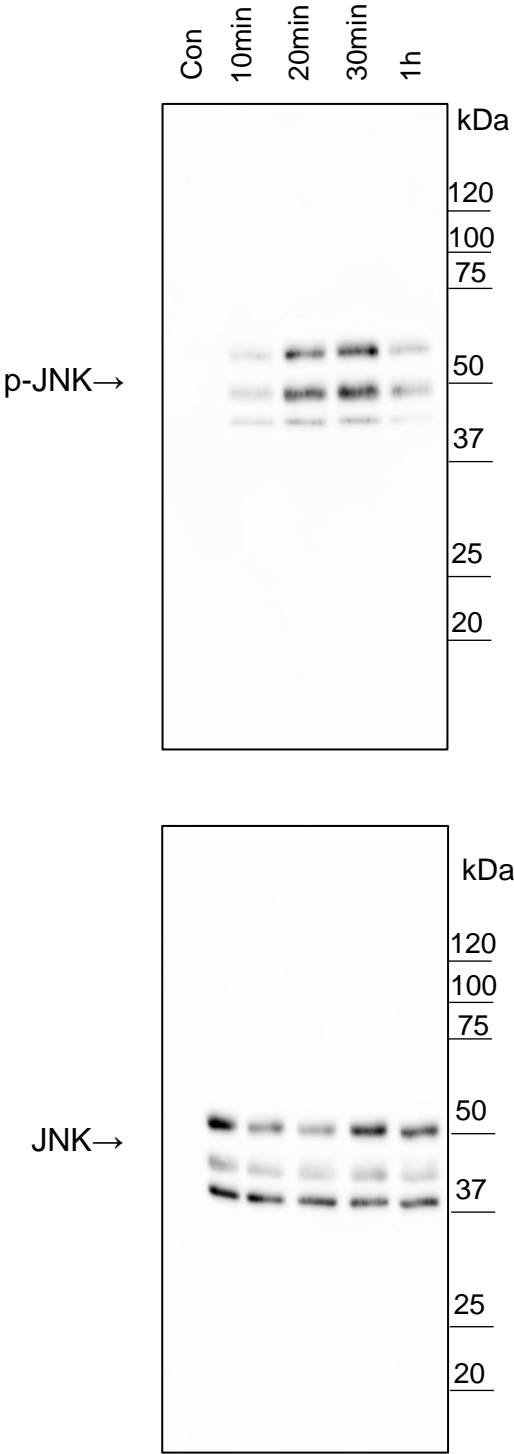

S2 Fig  
B

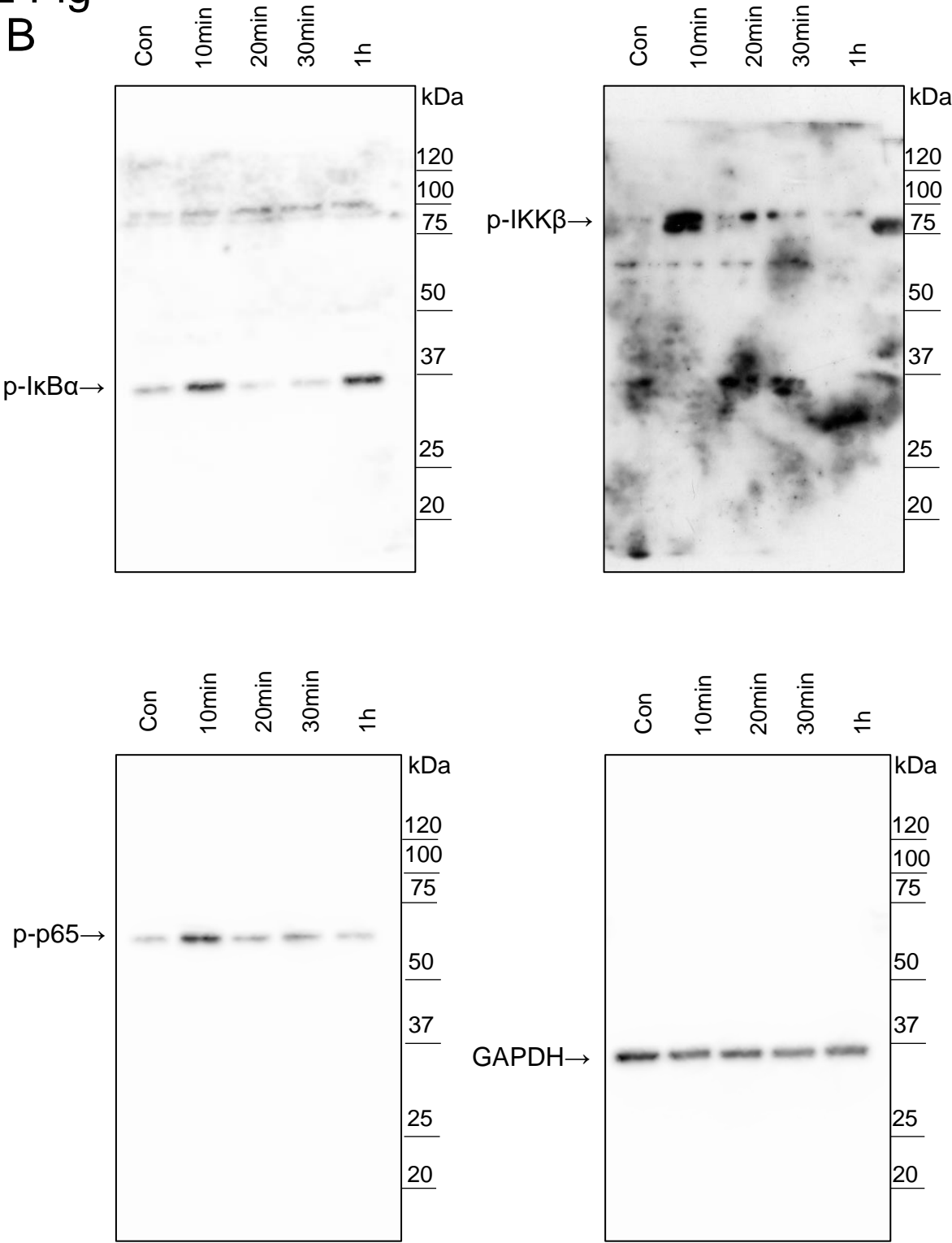

S2 Fig  
C

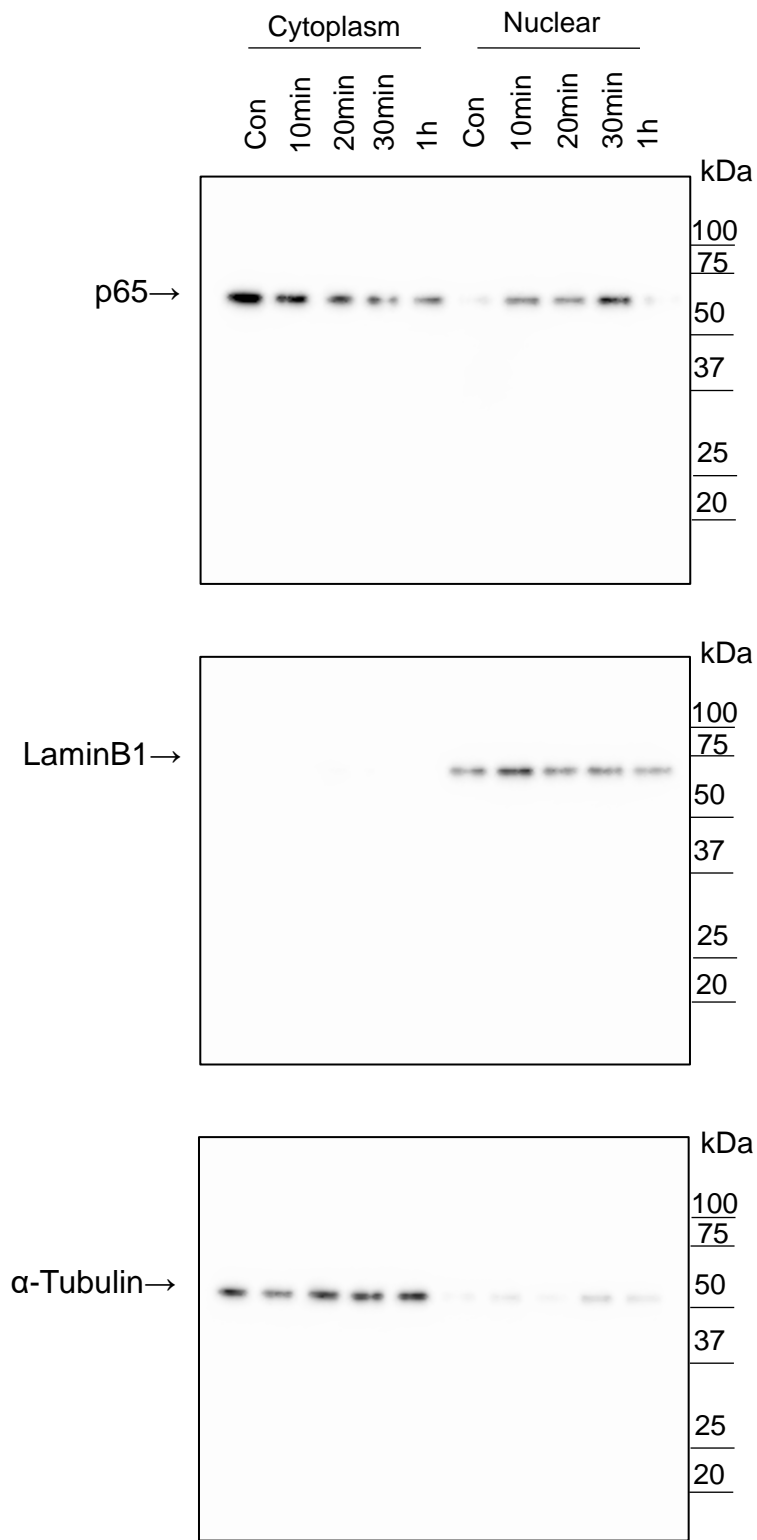

S2 Fig  
D

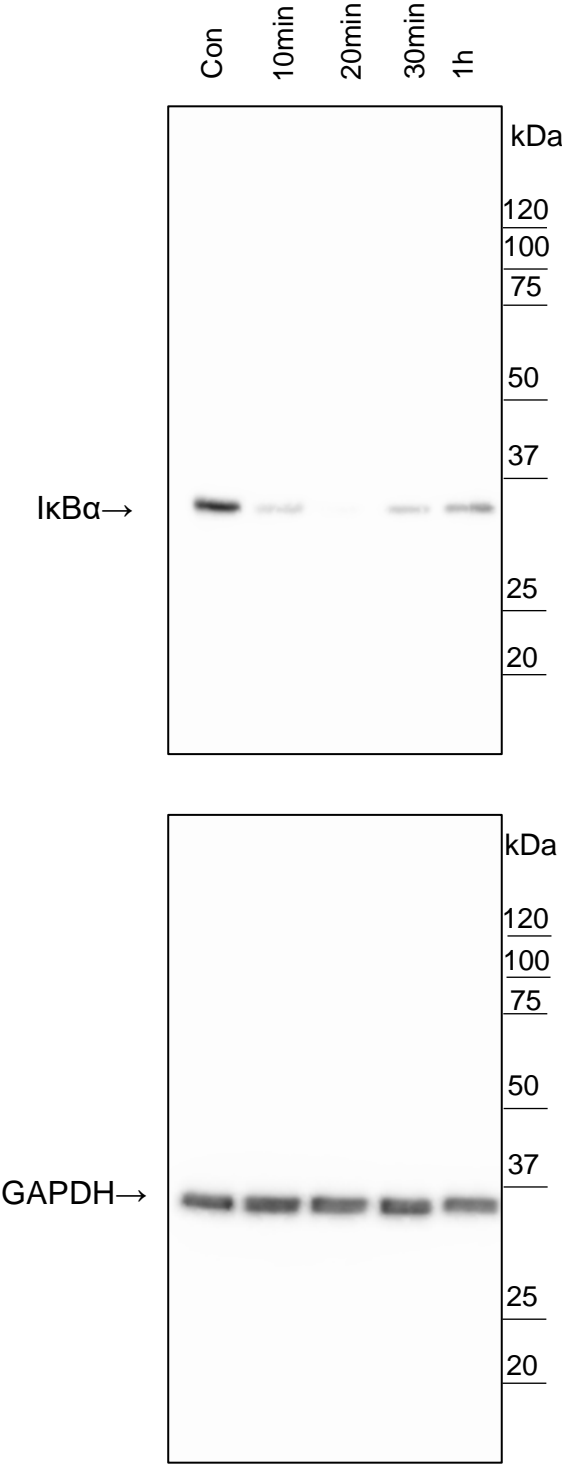

Supplement: S1 Raw images — (PDF) [file pone.0247840.s003.pdf]
